# Supplementary material for: Favorable Nonclinical Safety Profile of RSVpreF Bivalent Vaccine in Rats and Rabbits
Source: Vaccines (Basel). 2024 Dec 31;13(1):26. doi: 10.3390/vaccines13010026 (PMC11769190; doi:10.3390/vaccines13010026)
Supplement: Supplementary file 1 [file vaccines-13-00026-s001.zip › Supplemental Table S1_FC.pdf]

Supplemental Table S1. Effect of RSVpreF, with or without $Al(OH)_3$, on Food Consuption.

No statistical significance was observed at any period.

Dosing Days	Group	Male	Female
1-4	Saline	53.81 ± 5.68	38.44 ± 3.03
	Al[OH]3	55.65 ± 5.35	37.55 ± 3.86
	RSVpreF	54.78 ± 8.17	39.95 ± 5.53
	RSVpreF+Al(OH)3	57.33 ± 6.7	39.31 ± 6.89
4-8	Saline	83.68 ± 8.84	60.07 ± 5.74
	Al[OH]3	86.39 ± 8.35	60.53 ± 5.72
	RSVpreF	82.45 ± 5.55	61.84 ± 5.14
	RSVpreF+Al(OH)3	87.2 ± 6.82	61.35 ± 6.09
8-15	Saline	144.99 ± 10.49	100.12 ± 8.31
	Al[OH]3	146.86 ± 13.56	97.87 ± 4.64
	RSVpreF	140.92 ± 8.75	101.61 ± 10.31
	RSVpreF+Al(OH)3	146.44 ± 11.57	101.93 ± 7.04
15-22	Saline	146.74 ± 12.69	99.39 ± 6.09
	Al[OH]3	148.22 ± 12.16	99.98 ± 5.94
	RSVpreF	144.69 ± 9.57	104.55 ± 7.96
	RSVpreF+Al(OH)3	149.53 ± 9.76	102.2 ± 8.18
22-25	Saline	61.84 ± 5.71	40.83 ± 2.91
	Al[OH]3	60.53 ± 5.74	40.63 ± 2.84
	RSVpreF	59.67 ± 4.53	41.53 ± 2.76
	RSVpreF+Al(OH)3	60.82 ± 3.52	40.98 ± 3.13
25-29	Saline	80.43 ± 7.08	57.03 ± 5.84
	Al[OH]3	79.6 ± 6.07	56.64 ± 5.47
	RSVpreF	78.33 ± 5.44	57.7 ± 6.92
	RSVpreF+Al(OH)3	79.75 ± 5.22	55.85 ± 5.36
29-36	Saline	148.59 ± 12.13	102.13 ± 9.96
	Al[OH]3	146.83 ± 11.65	102.08 ± 8.5
	RSVpreF	144.97 ± 9.59	104.73 ± 9.88
	RSVpreF+Al(OH)3	147.43 ± 9.38	99.53 ± 8.06
1-36	Saline	897.15 ± 277.21	617.46 ± 180.93
	Al[OH]3	894.46 ± 268	614.08 ± 176.3
	RSVpreF	872 ± 238.82	627.66 ± 161.49
	RSVpreF+Al(OH)3	906.64 ± 282.31	621.08 ± 187.61
**Recovery Days**			
1-8	Saline	146.48 ± 15.42	97.22 ± 6.78
	Al[OH]3	140.02 ± 11.43	97.8 ± 3.85
	RSVpreF	135.2 ± 5.26	96.4 ± 7.64
	RSVpreF+Al(OH)3	144.26 ± 8.97	101.92 ± 10.36
8-15	Saline	144.5 ± 12.65	103.02 ± 5.83
	Al[OH]3	141.86 ± 12.15	102.96 ± 3.93
	RSVpreF	143.54 ± 5.98	101.46 ± 6.91
	RSVpreF+Al(OH)3	150.58 ± 4.49	103.14 ± 9.25
15-22	Saline	150.94 ± 13.52	100.94 ± 5.35
	Al[OH]3	143.88 ± 11.9	99.02 ± 3.97
	RSVpreF	137.4 ± 6.91	95.8 ± 7.73
	RSVpreF+Al(OH)3	149.7 ± 6.41	98.84 ± 8.65
22-26	Saline	89.26 ± 7.02	57.18 ± 3.94
	Al[OH]3	85.36 ± 9.06	56.64 ± 2.37
	RSVpreF	82.4 ± 6.16	53.56 ± 3.85
	RSVpreF+Al(OH)3	89.86 ± 5.36	55.88 ± 4.06
1-26	Saline	1264.24 ± 89.05	859.26 ± 58.99
	Al[OH]3	1246.54 ± 112.16	852.52 ± 24.51
	RSVpreF	1191.64 ± 45.76	838.98 ± 70.88
	RSVpreF+Al(OH)3	1287.36 ± 53.52	868.78 ± 71.1
