# Supplementary material for: Favorable Nonclinical Safety Profile of RSVpreF Bivalent Vaccine in Rats and Rabbits
Source: Vaccines (Basel). 2024 Dec 31;13(1):26. doi: 10.3390/vaccines13010026 (PMC11769190; doi:10.3390/vaccines13010026)
Supplement: Supplementary file 1 [file vaccines-13-00026-s001.zip › Supplemental Table S2_Clin Path.pdf]

Supplemental Table S2: Clinical Pathology.

Hematology Parameters.

Parameter	Description
HEMO	Hematology comment
RBC	Red Blood Cells
HGB	Hemoglobin
HCT	Hematocrit
MCV	Mean Cell Volume
MCH	Mean Cell Hemoglobin
MCHC	Mean Cell Hemoglobin Conc
RDW	Red Cell Distribution Width
RETIC_P	Reticulocyte
RETIC	Reticulocyte, Absolute
PLT	Platelets
MPV	Mean Platelet Volume
MORPH	Morphology
BURR	Burr Cells
WBC	White Blood Cells
NEUT	Neutrophil, Absolute
NEUT_P	Neutrophil
LYM	Lymphocyte, Absolute
LYM_P	Lymphocyte
MONO	Monocyte, Absolute
MONO_P	Monocyte
EO	Eosinophil, Absolute
EO_P	Eosinophil
BASO	Basophil, Absolute
BASO_P	Basophil
LUC	Large Unstained Cells, Absolute
LUC_P	Large Unstained Cells
COAG	Coagulation comment
PT	Prothrombin Time
APTT	Act. Partial Thromboplastin Time
FIB	Fibrinogen

Parameter	Day*	Saline		l[OH]3 Vehicle		RSVpreF + Al[OH]3		RSVpreF + Al[OH]3	
		Male	Female	Male	Female	Male	Female	Male	Female
BASO 10e3/uL	Day 3	0.01 ± 0	0.01 ± 0	0.01 ± 0	0.01 ± 0.01	0.01 ± 0	0 ± 0	0.01 ± 0	0 ± 0.01
	Day 38	0.01 ± 0.01	0 ± 0	0.01 ± 0.01	0 ± 0	0 ± 0.01	0 ± 0	0.01 ± 0.01	0 ± 0
	Recovery 28/Recovery 27	0.01 ± 0.01	0 ± 0	0 ± 0	0 ± 0	0 ± 0	0 ± 0	0 ± 0.01	0 ± 0
BASO.P	Day 3	0.09 ± 0.04	0.09 ± 0.04	0.11 ± 0.04	0.06 ± 0.05	0.09 ± 0.04	0.07 ± 0.08	0.09 ± 0.04	0.06 ± 0.05
	Day 38	0.11 ± 0.06	0.05 ± 0.05	0.12 ± 0.04	0.07 ± 0.07	0.09 ± 0.07	0.1 ± 0.05	0.11 ± 0.03	0.05 ± 0.05
	Recovery 28/Recovery 27	0.14 ± 0.05	0.02 ± 0.04	0.1 ± 0.07	0.12 ± 0.04	0.1 ± 0.07	0.1 ± 0.07	0.1 ± 0.07	0.1 ± 0.07
EO 10e3/uL	Day 3	0.16 ± 0.21	0.08 ± 0.02	0.14 ± 0.05	0.1 ± 0.04	0.1 ± 0.03	0.1 ± 0.03	0.11 ± 0.03	0.09 ± 0.02
	Day 38	0.09 ± 0.03	0.04 ± 0.03	0.11 ± 0.02	0.04 ± 0.01	0.09 ± 0.03	0.05 ± 0.02	0.13 ± 0.03	0.13 ± 0.05**
	Recovery 28/Recovery 27	0.06 ± 0.03	0.03 ± 0.01	0.07 ± 0.02	0.03 ± 0.01	0.07 ± 0.04	0.03 ± 0.01	0.05 ± 0.02	0.04 ± 0
EO.P	Day 3	1.73 ± 1.84	1.2 ± 0.36	1.49 ± 0.3	1.66 ± 0.68	1.26 ± 0.56	1.8 ± 0.55	1.21 ± 0.33	1.4 ± 0.43
	Day 38	1.47 ± 0.48	1.65 ± 0.58	1.73 ± 0.32	1.5 ± 0.41	1.74 ± 0.81	1.76 ± 0.61	1.88 ± 0.6	3.06 ± 1.18
	Recovery 28/Recovery 27	1.14 ± 0.34	1.82 ± 0.41	1.44 ± 0.39	1.96 ± 0.94	1.76 ± 0.79	1.94 ± 0.81	1.44 ± 0.48	1.76 ± 0.5
HCT %	Day 3	46.07 ± 0.76	43.04 ± 1.79	46.07 ± 1.55	43.21 ± 1.36	45.8 ± 1.06	42.7 ± 1.45	44.89 ± 1.61	42.7 ± 1.97
	Day 38	45.03 ± 1.61	41.78 ± 2.38	44.69 ± 2.18	41.33 ± 1.54	44.79 ± 1.47	40.91 ± 1.7	43.31 ± 2.09	40.52 ± 1.3
	Recovery 28/Recovery 27	47.76 ± 1.48	45.86 ± 2.04	46.2 ± 1.1	46.36 ± 1.43	46.42 ± 2.01	47.06 ± 0.92	45.46 ± 0.97	47.64 ± 4.81
HGB g/dL	Day 3	14.83 ± 0.24	14.36 ± 0.49	14.99 ± 0.5	14.37 ± 0.39	15.04 ± 0.41	14.13 ± 0.4	14.97 ± 0.81	14.16 ± 0.59
	Day 38	14.4 ± 0.61	13.57 ± 0.83	14.25 ± 0.58	13.53 ± 0.4	14.46 ± 0.28	13.31 ± 0.42	14.16 ± 0.71	13.17 ± 0.37
	Recovery 28/Recovery 27	15.16 ± 0.23	14.76 ± 0.36	14.84 ± 0.58	14.92 ± 0.28	14.6 ± 0.39	14.88 ± 0.24	14.5 ± 0.26	15.36 ± 1.38
LUC 10e3/uL	Day 3	0.05 ± 0.01	0.04 ± 0.01	0.08 ± 0.04	0.03 ± 0.01	0.05 ± 0.04	0.03 ± 0.02	0.06 ± 0.03	0.04 ± 0.02
	Day 38	0.04 ± 0.01	0.01 ± 0.01	0.04 ± 0.03	0.02 ± 0.01	0.03 ± 0.02	0.01 ± 0.01	0.04 ± 0.02	0.04 ± 0.02*
	Recovery 28/Recovery 27	0.02 ± 0.01	0 ± 0	0.01 ± 0.01	0.01 ± 0.01	0.01 ± 0*	0 ± 0.01	0.01 ± 0.01	0.01 ± 0
LUC.P	Day 3	0.61 ± 0.09	0.6 ± 0.15	0.81 ± 0.35	0.41 ± 0.17	0.54 ± 0.28	0.46 ± 0.16	0.67 ± 0.3	0.59 ± 0.21
	Day 38	0.72 ± 0.43	0.59 ± 0.27	0.68 ± 0.37	0.54 ± 0.27	0.46 ± 0.2	0.41 ± 0.17	0.59 ± 0.16	0.84 ± 0.34
	Recovery 28/Recovery 27	0.4 ± 0.16	0.18 ± 0.08	0.34 ± 0.13	0.36 ± 0.19	0.3 ± 0.07	0.3 ± 0.32	0.34 ± 0.13	0.3 ± 0.07
LYM 10e3/uL	Day 3	7.06 ± 1.27	5.73 ± 1.11	7.68 ± 1.66	4.77 ± 0.8	7.19 ± 2.74	4.86 ± 1.35	6.97 ± 1.06	5.13 ± 1.58
	Day 38	4.57 ± 1.39	1.89 ± 0.81	4.72 ± 1.24	2.11 ± 0.8	4.27 ± 1.51	2.08 ± 0.89	4.72 ± 1.53	2.58 ± 0.43
	Recovery 28/Recovery 27	4.2 ± 1.98	1.38 ± 0.28	3.63 ± 1.02	1.52 ± 0.23	3.16 ± 0.71	1.54 ± 0.6	2.9 ± 0.56	2.04 ± 0.65

Parameter	Day*	Saline		l[OH]3 Vehicle		RSVpreF + Al[OH]3		RSVpreF + Al[OH]3	
		Male	Female	Male	Female	Male	Female	Male	Female
LYM.P	Day 3	83.01 ± 3.06	85.71 ± 1.68	80.36 ± 4.56	76.79 ± 6.83	79.49 ± 7.69	81.29 ± 3.06	76.23 ± 2.59	79.96 ± 2.57
	Day 38	77.3 ± 2.98	76.77 ± 5.11	75.95 ± 3.02	73.58 ± 7.16	72.95 ± 12.93	74.88 ± 5.7	68.28 ± 5.09	63.19 ± 7.74
	Recovery 28/Recovery 27	76.62 ± 5.83	72.28 ± 4.74	78.86 ± 5.2	78.2 ± 4.44	73.08 ± 3.86	78.32 ± 6.5	77.12 ± 8.15	78.38 ± 4.37
MCH pg	Day 3	18.73 ± 0.64	19.1 ± 0.44	18.91 ± 0.67	19.47 ± 0.81	18.9 ± 0.81	18.84 ± 0.81	19.51 ± 0.96	19.63 ± 0.69
	Day 38	17.98 ± 0.64	18.64 ± 0.3	17.92 ± 0.67	19 ± 0.72	18.02 ± 0.9	18.55 ± 1.01	18.48 ± 1.04	18.96 ± 0.59
	Recovery 28/Recovery 27	17.8 ± 0.78	19.24 ± 0.65	17.96 ± 0.88	19.1 ± 0.44	17.46 ± 0.62	18.34 ± 0.23	17.8 ± 0.25	19.02 ± 0.77
MCHC g/dL	Day 3	32.17 ± 0.26	33.34 ± 0.67	32.51 ± 0.29	33.23 ± 0.42	32.86 ± 0.77**	33.09 ± 0.42	33.33 ± 0.69*	33.13 ± 0.77
	Day 38	31.98 ± 0.96	32.45 ± 0.44	31.91 ± 0.65	32.73 ± 0.88	32.3 ± 0.74	32.54 ± 0.87	32.63 ± 0.79	32.52 ± 0.73
	Recovery 28/Recovery 27	31.76 ± 0.62	32.16 ± 1.11	32.2 ± 1.17	32.26 ± 1.05	31.48 ± 0.67	31.6 ± 0.59	31.94 ± 0.42	32.22 ± 1.12
MCV fL	Day 3	58.17 ± 1.51	57.27 ± 0.71	58.14 ± 1.9	58.56 ± 2.35	57.51 ± 1.52	56.97 ± 1.96	58.59 ± 2.41	59.24 ± 1.66
	Day 38	56.21 ± 1.26	57.45 ± 0.6	56.1 ± 1.7	58.25 ± 1.96	55.76 ± 1.79	56.67 ± 1.49	56.5 ± 3.01	58.35 ± 1.86
	Recovery 28/Recovery 27	55.96 ± 1.78	59.92 ± 1.78	55.6 ± 1.27	59.32 ± 2.38	55.48 ± 1.72	58.08 ± 0.99	55.8 ± 0.75	59.02 ± 1.68
MONO 10e3/uL	Day 3	0.17 ± 0.07	0.13 ± 0.03	0.25 ± 0.08	0.17 ± 0.03	0.18 ± 0.05	0.12 ± 0.05	0.25 ± 0.06	0.17 ± 0.06
	Day 38	0.11 ± 0.04	0.05 ± 0.02	0.13 ± 0.05	0.08 ± 0.03	0.11 ± 0.02	0.05 ± 0.03	0.14 ± 0.02	0.11 ± 0.04
	Recovery 28/Recovery 27	0.1 ± 0.04	0.04 ± 0.02	0.1 ± 0.04	0.04 ± 0.01	0.09 ± 0.04	0.04 ± 0.02	0.07 ± 0.02	0.07 ± 0.03
MONO.P	Day 3	2.11 ± 1.1	1.93 ± 0.44	2.6 ± 0.79	2.84 ± 0.4	2.13 ± 0.53	1.91 ± 0.44	2.71 ± 0.46	2.69 ± 0.63
	Day 38	1.87 ± 0.69	1.9 ± 0.28	2.09 ± 0.48	2.91 ± 0.5	2.04 ± 0.57	1.85 ± 0.3	2.11 ± 0.5	2.58 ± 0.63
	Recovery 28/Recovery 27	1.94 ± 0.47	2.2 ± 1.06	2.08 ± 0.57	2.16 ± 0.56	2 ± 0.55	2.14 ± 0.77	1.88 ± 0.29	2.66 ± 0.43
MPV fL	Day 3	7.91 ± 0.3	7.41 ± 0.31	7.84 ± 0.47	7.59 ± 0.22	7.8 ± 0.31	7.81 ± 0.23	7.86 ± 0.39	7.71 ± 0.45
	Day 38	8.54 ± 0.21	8.17 ± 0.21	8.71 ± 0.49	8.32 ± 0.34	8.64 ± 0.32	8.48 ± 0.28	8.76 ± 0.29	8.3 ± 0.37
	Recovery 28/Recovery 27	9.18 ± 0.47	9.82 ± 0.37	9.06 ± 0.38	9.8 ± 0.58	8.7 ± 0.31	9.52 ± 0.52	9.36 ± 0.42	9.66 ± 0.27
NEUT 10e3/uL	Day 3	1.06 ± 0.26	0.68 ± 0.09	1.36 ± 0.38	1.12 ± 0.42	1.28 ± 0.32	0.84 ± 0.22	1.76 ± 0.44	0.95 ± 0.17
	Day 38	1.11 ± 0.4	0.45 ± 0.2	1.18 ± 0.28	0.58 ± 0.18	1.18 ± 0.28	0.56 ± 0.19	1.85 ± 0.57*	1.25 ± 0.39**
	Recovery 28/Recovery 27	0.97 ± 0.18	0.46 ± 0.2	0.75 ± 0.13	0.35 ± 0.16	0.96 ± 0.11	0.31 ± 0.07	0.74 ± 0.36	0.43 ± 0.17
NEUT.P	Day 3	12.44 ± 1.8	10.46 ± 1.5	14.63 ± 4.54	18.24 ± 6.61	16.49 ± 7.07	14.44 ± 3.3	19.09 ± 2.87	15.3 ± 2.71
	Day 38	18.52 ± 2.84	19.04 ± 5.3	19.41 ± 2.89	21.4 ± 7.18	22.74 ± 11.9	20.99 ± 6.18	27.04 ± 5.18	30.26 ± 7.08
	Recovery 28/Recovery 27	19.76 ± 5.75	23.46 ± 5.97	17.2 ± 5.18	17.2 ± 4.77	22.8 ± 3.93	17.24 ± 6.01	19.08 ± 7.64	16.78 ± 4.9

Parameter	Day*	Saline		l[OH]3 Vehicle		RSVpreF + Al[OH]3		RSVpreF + Al[OH]3	
		Male	Female	Male	Female	Male	Female	Male	Female
PLT 10e3/uL	Day 3	917.29 ± 121.05	898.86 ± 75.63	866 ± 93.79	838.57 ± 56.42	842.14 ± 138.71	844.86 ± 101.95	855.14 ± 111.16	806 ± 121.74
	Day 38	882.9 ± 101.56	870.5 ± 118.24	842.2 ± 79.09	905 ± 97.29	870.2 ± 62.25	809.1 ± 87.05	873.33 ± 93.69	852.9 ± 124.16
	Recovery 28/Recovery 27	815.2 ± 65.67	756.6 ± 39.51	839.8 ± 62.45	809.2 ± 96.54	827 ± 132.47	828 ± 131.65	717.2 ± 67.87	766.8 ± 139.78
RBC 10e6/uL	Day 3	7.92 ± 0.27	7.51 ± 0.26	7.93 ± 0.32	7.4 ± 0.49	7.97 ± 0.27	7.51 ± 0.41	7.67 ± 0.28	7.22 ± 0.41
	Day 38	8.02 ± 0.36	7.27 ± 0.41	7.97 ± 0.43	7.13 ± 0.37	8.04 ± 0.42	7.18 ± 0.44	7.66 ± 0.3	6.95 ± 0.3
	Recovery 28/Recovery 27	8.54 ± 0.5	7.66 ± 0.22	8.28 ± 0.24	7.82 ± 0.13	8.36 ± 0.33	8.1 ± 0.14	8.15 ± 0.21	8.07 ± 0.71
RDW %	Day 3	12.44 ± 0.46	11.99 ± 0.68	12.39 ± 0.12	12.03 ± 0.58	12.67 ± 0.55	12.36 ± 0.56	12.39 ± 0.6	12.33 ± 0.55
	Day 38	11.77 ± 0.36	10.76 ± 0.26	11.61 ± 0.32	11.26 ± 0.72	12.1 ± 0.58	11.16 ± 0.46	11.61 ± 0.46	11.02 ± 0.5
	Recovery 28/Recovery 27	12.6 ± 0.34	11.15 ± 0.21	12.05 ± 0.31	11.52 ± 0.4	12.34 ± 0.34	11.3 ± 0.25	12.5 ± 0.34	11.66 ± 0.82
RETIC 10e3/uL	Day 3	364.14 ± 48.75	311.29 ± 39.69	383.43 ± 41.72	331 ± 51.96	364.71 ± 51.89	346.57 ± 50.66	341.57 ± 39.16	361.57 ± 63.23
	Day 38	225.6 ± 15.76	236.5 ± 38.75	233.8 ± 24.55	266.78 ± 57.94	206 ± 26.11	243.7 ± 28.53	228.11 ± 25.87	267.7 ± 31.56
	Recovery 28/Recovery 27	196.8 ± 21.16	144 ± 22.7	159.2 ± 22.71	155 ± 27.21	182.2 ± 17.34	139.4 ± 26.82	179.2 ± 9.98	135.4 ± 36.74
RETIC.P	Day 3	4.61 ± 0.73	4.16 ± 0.63	4.86 ± 0.67	4.47 ± 0.58	4.57 ± 0.62	4.64 ± 0.79	4.47 ± 0.65	5.01 ± 0.84
	Day 38	2.82 ± 0.23	3.26 ± 0.54	2.94 ± 0.33	3.74 ± 0.8	2.57 ± 0.37	3.4 ± 0.44	2.98 ± 0.33	3.85 ± 0.4
	Recovery 28/Recovery 27	2.3 ± 0.14	1.88 ± 0.31	1.92 ± 0.25	1.98 ± 0.33	2.18 ± 0.16	1.72 ± 0.33	2.2 ± 0.14	1.66 ± 0.31
WBC 10e3/uL	Day 3	8.51 ± 1.56	6.66 ± 1.21	9.5 ± 1.78	6.2 ± 0.76	8.81 ± 3.06	5.97 ± 1.58	9.14 ± 1.43	6.37 ± 1.78
	Day 38	5.91 ± 1.79	2.44 ± 0.97	6.19 ± 1.53	2.84 ± 0.94	5.69 ± 1.53	2.74 ± 1.08	6.89 ± 1.96	4.11 ± 0.65*
	Recovery 28/Recovery 27	5.36 ± 2.07	1.92 ± 0.44	4.56 ± 1.08	1.96 ± 0.38	4.28 ± 0.8	1.94 ± 0.65	3.78 ± 0.79	2.6 ± 0.77

*Male samples were collected on Recovery Day 28 while Female samples were collected on Recovery Day 27; *p<0.05; *p<0.01.

Coagulation parameters

Parameter	Day*	Saline		l[OH]3 Vehicle		RSVpreF + Al[OH]3		RSVpreF + Al[OH]3	
		Male	Female	Male	Female	Male	Female	Male	Female
APTT sec	Day 38	17.67 ± 0.96	16.78 ± 0.55	17.4 ± 0.92	16.91 ± 0.96	17.3 ± 0.88	16.43 ± 0.91	17.01 ± 1.88	16.61 ± 0.67
	Recovery 28/Recovery 27	17.74 ± 1.15	16.82 ± 0.58	18.56 ± 1.1	16.28 ± 1.16	16.92 ± 2.03	16.86 ± 0.38	18.42 ± 1.48	16.8 ± 0.67
FIB	Day 38	267.4 ± 17.29	207.7 ± 16.55	266.7 ± 22.54	209.56 ± 17.85	267.9 ± 12.9	202.6 ± 9.99	361.4 ± 24.25**	341 ± 60.69

Parameter	Day*	Saline		l[OH]3 Vehicle		RSVpreF + Al[OH]3		RSVpreF + Al[OH]3	
		Male	Female	Male	Female	Male	Female	Male	Female
mg/dL	Recovery 28/Recovery 27	258.6 ± 20.01	199.2 ± 24.13	264.8 ± 14.92	181.4 ± 16.68	255.8 ± 22.21	215 ± 16.84	257 ± 19.3	207.6 ± 33.84
PT sec	Day 38	15.36 ± 1.44	12.62 ± 0.61	14.34 ± 1.4	12.69 ± 0.81	15.37 ± 2.41	12.63 ± 0.59	14.61 ± 0.91	12.65 ± 0.76
	Recovery 28/Recovery 27	15.24 ± 1.79	11.52 ± 0.79	16.44 ± 2.99	11.48 ± 0.41	15.02 ± 2.9	11.56 ± 0.38	17.6 ± 1.66	11.46 ± 0.81

*Male samples were collected on Recovery Day 28 while Female samples were collected on Recovery Day 27; *p<0.05; *p<0.01.

Clinical Chemistry Parameters:

Parameter	Description
ALT	Alanine Aminotransferase
AST	Aspartate Aminotransferase
ALP	Alkaline Phosphatase
GGT	Gamma Glutamyl Transferase
CK	Creatine Kinase
TBIL	Bilirubin, Total
CHOL	Cholesterol
TRIG	Triglycerides
GLUC	Glucose
TP	Protein, Total
ALB	Albumin
GLOB	Globulin
AG	Albumin/Globulin Ratio
BUN	Blood Urea Nitrogen
CREA	Creatinine
PHOS	Phosphorus
CA	Calcium
NA	Sodium
K	Potassium
CL	Chloride
A2M	Alpha-2-Macroglobulin
HEM_IND	Hemolytic Index
ICT_IND	Icterus Index
LIP_IND	Lipemic Index

Parameter	Day*	Saline		l[OH]3 Vehicle		RSVpreF + Al[OH]3		RSVpreF + Al[OH]3	
		Male	Female	Male	Female	Male	Female	Male	Female
A2M ug/mL	Day 3	13.88 ± 4.26	10.5 ± 1.6	15.88 ± 3	10.62 ± 2.2	21.88 ± 23.8	10.62 ± 1.19	13.62 ± 6.3	12.12 ± 2.53
	Day 22	9.53 ± 2.13	11.87 ± 2.53	10.27 ± 2.31	12 ± 2.67	10.33 ± 2.87	11.8 ± 1.61	9.53 ± 3.16	12.13 ± 2.26
	Day 38	10.3 ± 2.26	10.4 ± 2.07	9 ± 2.11	10.6 ± 2.95	8.3 ± 1.42	13.3 ± 1.95	10.4 ± 4.5	22.9 ± 8.12**
	Recovery 28/Recovery 27	4.8 ± 0.84	8.2 ± 1.3	6.4 ± 0.89	7.8 ± 1.48	5.6 ± 1.14	9.4 ± 1.95	4.8 ± 1.64	8.4 ± 1.95
A/G	Day 3	1.64 ± 0.07	1.57 ± 0.1	1.61 ± 0.06	1.6 ± 0.08	1.62 ± 0.09	1.57 ± 0.07	1.65 ± 0.08	1.59 ± 0.06
	Day 38	1.65 ± 0.07	1.62 ± 0.08	1.66 ± 0.11	1.67 ± 0.11	1.66 ± 0.07	1.64 ± 0.07	1.52 ± 0.08**	1.52 ± 0.06**
	Recovery 28/Recovery 27	1.62 ± 0.13	1.62 ± 0.08	1.58 ± 0.08	1.56 ± 0.05	1.6 ± 0.07	1.56 ± 0.05	1.48 ± 0.04	1.48 ± 0.08
ALB g/dL	Day 3	3.9 ± 0.13	4.2 ± 0.13	3.85 ± 0.11	4.18 ± 0.15	3.88 ± 0.09	3.99 ± 0.1**	3.89 ± 0.11	4.18 ± 0.1
	Day 38	3.67 ± 0.13	3.98 ± 0.25	3.68 ± 0.11	3.88 ± 0.22	3.64 ± 0.11	3.81 ± 0.15	3.57 ± 0.05*	3.82 ± 0.13
	Recovery 28/Recovery 27	3.56 ± 0.09	3.92 ± 0.08	3.48 ± 0.13	3.82 ± 0.16	3.52 ± 0.16	3.78 ± 0.04	3.44 ± 0.09	3.86 ± 0.05
ALP	Day 3	151 ± 35.58	67.62 ± 21.95	130.25 ± 27.97	62.12 ± 15.42	138.38 ± 24.55	58.38 ± 20.25	122.25 ± 17.3	52.12 ± 9.14

Parameter	Day*	Saline		l[OH]3 Vehicle		RSVpreF + Al[OH]3		RSVpreF + Al[OH]3	
		Male	Female	Male	Female	Male	Female	Male	Female
U/L	Day 38	70.8 ± 12.67	32.5 ± 9.38	76.6 ± 14.33	33.6 ± 13.87	72.4 ± 13.43	29.9 ± 6.33	59.5 ± 11.6*	31.6 ± 5.21
	Recovery 28/Recovery 27	66.4 ± 17.36	30.4 ± 4.04	60.8 ± 10.33	29.2 ± 11.3	75.2 ± 11.03	29.4 ± 7.16	56.4 ± 6.43	29 ± 10.61
ALT U/L	Day 3	25.38 ± 3.85	23.12 ± 4.67	27 ± 4.93	29 ± 17.48	24.5 ± 3.89	24.25 ± 9.05	26.62 ± 4.27	29.12 ± 11.41
	Day 38	20.8 ± 3.01	15.9 ± 3.98	21.5 ± 3.1	17.8 ± 2.25	19.8 ± 2.15	16.9 ± 2.85	22.6 ± 3.27	19.1 ± 7.56
	Recovery 28/Recovery 27	18.2 ± 3.03	16.6 ± 0.89	18.8 ± 1.92	15.4 ± 1.67	19.6 ± 2.3	13.2 ± 1.1*	19.4 ± 2.51	16 ± 3.32
AST U/L	Day 3	91.5 ± 11.7	89.75 ± 11.22	93.12 ± 9.88	87.62 ± 17.04	89.88 ± 12.15	82 ± 5.9	92 ± 10.97	85.5 ± 17.62
	Day 38	81.2 ± 15.03	73.2 ± 12.96	81.2 ± 12.35	77 ± 11.49	80.7 ± 15.92	75.4 ± 15.42	83.5 ± 10.76	76.8 ± 12.84
	Recovery 28/Recovery 27	84.8 ± 18.57	83.4 ± 9.42	82.6 ± 7.99	73.2 ± 10.18	83 ± 21.67	66.6 ± 5.41*	77.2 ± 9.01	82.6 ± 23.48
BUN mg/dL	Day 3	19.5 ± 3.51	18.88 ± 2.23	18.38 ± 2.56	21.25 ± 4.89	17.88 ± 2.95	17.62 ± 1.77	18.38 ± 2.26	19.88 ± 3
	Day 38	18.6 ± 2.72	20.3 ± 4.11	18.9 ± 2.92	19.1 ± 3.67	18.9 ± 2.47	20.7 ± 2.41	18.8 ± 2.97	20.5 ± 3.41
	Recovery 28/Recovery 27	16 ± 1.58	23.8 ± 3.27	17.6 ± 3.29	20.8 ± 2.95	18.4 ± 4.34	21.6 ± 4.16	16.8 ± 1.64	21.6 ± 3.36
CA mg/dL	Day 3	10.29 ± 0.2	10.3 ± 0.19	10.28 ± 0.21	10.18 ± 0.13	10.2 ± 0.32	10 ± 0.36	10.18 ± 0.32	10.06 ± 0.19
	Day 38	9.98 ± 0.21	10.23 ± 0.31	9.96 ± 0.27	10.31 ± 0.37	10.02 ± 0.29	10 ± 0.28	9.98 ± 0.37	10.23 ± 0.29
	Recovery 28/Recovery 27	9.5 ± 0.4	9.66 ± 0.5	9.56 ± 0.44	9.58 ± 0.08	9.38 ± 0.33	9.64 ± 0.11	9.3 ± 0.29	9.76 ± 0.18
CHOL mg/dL	Day 3	62.12 ± 8.61	53.38 ± 15.28	56.88 ± 8.27	60.62 ± 8.63	64.75 ± 9.1	52.5 ± 8.02	58.62 ± 12.68	52 ± 13.16
	Day 38	60.3 ± 7.48	43.5 ± 15.07	56.7 ± 6.34	47.2 ± 10.75	61.9 ± 9.28	39.6 ± 9.96	66 ± 12.02	47.5 ± 10.99
	Recovery 28/Recovery 27	58.2 ± 7.4	39.8 ± 16.08	51.2 ± 11.41	44 ± 10.05	53.4 ± 7.3	38.6 ± 7.54	49 ± 3.54	43.6 ± 17.44
CK U/L	Day 3	884.5 ± 264.47	769.12 ± 227.6	881.12 ± 107.26	719.25 ± 353.9	796.62 ± 177.59	661.25 ± 136.51	781.75 ± 266.79	646.38 ± 134.23
	Day 22	867.53 ± 221.78	631.07 ± 196.97	561.53 ± 155.88	511.8 ± 149.33	679.13 ± 302.38*	642.8 ± 312.16	608.87 ± 190.73	514.87 ± 236.27
	Day 38	605.4 ± 397.63	504.2 ± 284.6	546 ± 212.28	639.2 ± 492.94	603.9 ± 299.01	459.2 ± 287.8	621.2 ± 335.47	417.2 ± 241.58
	Recovery 28/Recovery 27	809.6 ± 308.61	639.2 ± 158	704.6 ± 108.97	532.6 ± 172.45	713.8 ± 200.74	399.4 ± 150.34	565.2 ± 247.07	564.4 ± 235.96
CL mmol/L	Day 3	102.88 ± 0.99	104 ± 1.2	102.75 ± 1.49	104.12 ± 1.55	102.88 ± 1.73	104.62 ± 1.51	103.38 ± 1.19	105 ± 2.07
	Day 38	107.3 ± 1.57	108 ± 0.67	106.4 ± 1.17	107 ± 1.41	106.7 ± 1.89	107.8 ± 1.69	106.8 ± 0.92	108.3 ± 1.49
	Recovery 28/Recovery 27	105.8 ± 0.84	107.2 ± 1.3	105.6 ± 1.82	107 ± 2.35	106 ± 0.71	107.4 ± 1.82	107.8 ± 1.64	108.4 ± 2.88
CREA mg/dL	Day 3	0.29 ± 0.04	0.35 ± 0.05	0.3 ± 0.05	0.35 ± 0.05	0.28 ± 0.05	0.31 ± 0.04	0.31 ± 0.04	0.35 ± 0.05
	Day 38	0.29 ± 0.03	0.33 ± 0.05	0.29 ± 0.03	0.33 ± 0.05	0.27 ± 0.05	0.38 ± 0.06	0.27 ± 0.05	0.33 ± 0.05

Parameter	Day*	Saline		l[OH]3 Vehicle		RSVpreF + Al[OH]3		RSVpreF + Al[OH]3	
		Male	Female	Male	Female	Male	Female	Male	Female
	Recovery 28/Recovery 27	0.28 ± 0.04	0.38 ± 0.04	0.26 ± 0.05	0.34 ± 0.05	0.26 ± 0.05	0.32 ± 0.04	0.3 ± 0	0.36 ± 0.05
GLOB g/dL	Day 3	2.38 ± 0.13	2.67 ± 0.13	2.4 ± 0.08	2.62 ± 0.07	2.4 ± 0.15	2.55 ± 0.13	2.36 ± 0.13	2.62 ± 0.14
	Day 38	2.24 ± 0.1	2.46 ± 0.16	2.24 ± 0.13	2.33 ± 0.18	2.2 ± 0.11	2.33 ± 0.11	2.35 ± 0.13	2.53 ± 0.13**
	Recovery 28/Recovery 27	2.22 ± 0.16	2.44 ± 0.13	2.2 ± 0.07	2.42 ± 0.04	2.22 ± 0.13	2.42 ± 0.08	2.3 ± 0.07	2.64 ± 0.11*
GLUC mg/dL	Day 3	97.38 ± 16.45	90.38 ± 19.35	101.38 ± 18.02	92.5 ± 13.21	96.5 ± 18.17	93.12 ± 17.22	89 ± 14.26	92.5 ± 13.05
	Day 38	144.6 ± 37.45	115.8 ± 27	137.3 ± 24.83	114.1 ± 21.06	141 ± 22.87	118 ± 15.54	125 ± 19.7	120 ± 13.16
	Recovery 28/Recovery 27	160.8 ± 34.47	95 ± 10.58	149.8 ± 21.75	100 ± 4.42	155.6 ± 31.81	109.8 ± 15.93	139.2 ± 17.77	102.2 ± 18.86
K mmol/L	Day 3	4.75 ± 0.22	4.31 ± 0.24	4.82 ± 0.17	4.24 ± 0.17	4.74 ± 0.19	4.09 ± 0.24	4.4 ± 0.18**	4.06 ± 0.22
	Day 38	4.18 ± 0.23	3.83 ± 0.33	3.97 ± 0.23	3.87 ± 0.37	4.08 ± 0.2	3.62 ± 0.37	4.27 ± 0.25*	3.79 ± 0.16
	Recovery 28/Recovery 27	4.42 ± 0.28	3.7 ± 0.23	4.14 ± 0.27	3.82 ± 0.51	4.04 ± 0.23	3.7 ± 0.16	4.08 ± 0.19	3.86 ± 0.4
NA. mmol/L	Day 3	143.38 ± 0.52	143.75 ± 0.89	142.75 ± 0.89	141.75 ± 0.89	143.12 ± 0.83	142.38 ± 0.74*	143.38 ± 0.92	142 ± 1.2
	Day 38	144.3 ± 1.83	142.6 ± 0.84	143.9 ± 0.88	142.2 ± 1.23	144.2 ± 0.92	143 ± 1.49	143.5 ± 0.53	142.5 ± 0.71
	Recovery 28/Recovery 27	143 ± 0.71	143 ± 1.41	143 ± 0.71	143 ± 1	143.4 ± 1.14	143 ± 1.73	143.8 ± 1.1	143.2 ± 1.1
PHOS mg/dL	Day 3	7.84 ± 0.56	6.29 ± 0.85	7.61 ± 0.33	6.03 ± 0.53	7.7 ± 0.76	5.7 ± 0.54	7.66 ± 0.51	5.29 ± 0.39*
	Day 38	6.51 ± 0.63	6.15 ± 1.15	6.57 ± 0.7	5.9 ± 0.86	6.61 ± 0.85	5.84 ± 1.37	6.78 ± 0.63	5.74 ± 0.76
	Recovery 28/Recovery 27	5.6 ± 0.62	5.24 ± 0.59	6.18 ± 0.72	5.88 ± 0.63	6.18 ± 0.73	5.72 ± 0.44	5.84 ± 0.48	5.92 ± 0.58
TP g/dL	Day 3	6.28 ± 0.24	6.88 ± 0.17	6.25 ± 0.16	6.8 ± 0.19	6.28 ± 0.21	6.54 ± 0.2*	6.25 ± 0.21	6.8 ± 0.22
	Day 38	5.91 ± 0.18	6.44 ± 0.38	5.92 ± 0.19	6.21 ± 0.36	5.84 ± 0.19	6.14 ± 0.24	5.92 ± 0.15	6.35 ± 0.21
	Recovery 28/Recovery 27	5.78 ± 0.22	6.36 ± 0.21	5.68 ± 0.15	6.24 ± 0.21	5.74 ± 0.27	6.2 ± 0.12	5.74 ± 0.13	6.5 ± 0.1
TRIG mg/dL	Day 3	57.88 ± 13.23	31.5 ± 13.23	72.38 ± 17.33	38 ± 13.95	58.38 ± 13.3	40.38 ± 10	51.75 ± 15.1	41 ± 10.7
	Day 38	47 ± 20.41	28.3 ± 7.75	43.4 ± 14.29	32.2 ± 8.69	44.2 ± 15.85	29.5 ± 7.65	35.7 ± 12.75	28.7 ± 6.02
	Recovery 28/Recovery 27	54.4 ± 16.71	22.4 ± 3.13	43.2 ± 13.14	29.8 ± 9.88	39.6 ± 13.89	30.4 ± 4.93	25.8 ± 7.79	26.6 ± 4.56

Male samples were collected on Recovery Day 28 while Female samples were collected on Recovery Day 27; *p<0.05; p<0.01.

Urinalysis parameters:

Parameter	Description
COLOR	Color
CLARITY	Clarity
SG	Specific Gravity
VOLUME	Total Volume
PH	pH
GLU	Glucose Urine
KETONE	Ketones
PRO	Protein
BIL	BilirubIn
BLOOD	Blood
F_ELEM	Formed Elements
U_RBC	RBC, Urine
U_WBC	WBC, Urine
NON_SQ	Epithelial Cells, Non-Squamous
SQ_EPI	Epithelial Cells, Squamous
AMORPH	Crystal, Amorphous
TR_PHOS	Crystal, Triple Phosphate
SPERM	Sperm
BACT	Bacteria

Parameter	Day[*]	Saline		I[OH]3 Vehicle		RSVpreF + Al[OH]3		RSVpreF + Al[OH]3	
		Male	Female	Male	Female	Male	Female	Male	Female
SG	Day 38	1.04 ± 0.02	1.03 ± 0.02	1.03 ± 0.02	1.04 ± 0.02	1.03 ± 0.02	1.04 ± 0.01	1.03 ± 0.02	1.05 ± 0.02
	Recovery 28/Recovery 27	1.03 ± 0.02	1.05 ± 0.01	1.03 ± 0.02	1.03 ± 0.02	1.04 ± 0.02	1.04 ± 0.01	1.03 ± 0.02	1.04 ± 0.02
VOLUME	Day 38	8.95 ± 7.62	8.95 ± 8.19	14.55 ± 11.88	4.85 ± 2.87	11.4 ± 9.02	3.75 ± 1.81	10.9 ± 12.16	3.15 ± 1.73
	Recovery 28/Recovery 27	14.8 ± 12.93	2.8 ± 1.3	11.2 ± 7.09	7.4 ± 6.11	6.6 ± 4.51	5 ± 2.83	16.2 ± 19.49	5.4 ± 4.28
pH	Day 38	6.85 ± 0.34	6.2 ± 0.48	6.8 ± 0.35	6.2 ± 0.42	6.9 ± 0.46	5.95 ± 0.44	6.85 ± 0.47	6.15 ± 0.41
	Recovery 28/Recovery 27	7 ± 0.35	5.8 ± 0.27	6.9 ± 0.22	6.4 ± 0.42	6.8 ± 0.27	6.3 ± 0.27	7 ± 0.35	6 ± 0.35

[*]Male samples were collected on Recovery Day 28 while Female samples were collected on Recovery Day 27;
