# Supplementary material for: Favorable Nonclinical Safety Profile of RSVpreF Bivalent Vaccine in Rats and Rabbits
Source: Vaccines (Basel). 2024 Dec 31;13(1):26. doi: 10.3390/vaccines13010026 (PMC11769190; doi:10.3390/vaccines13010026)
Supplement: Supplementary file 1 [file vaccines-13-00026-s001.zip › Supplemental Table S3_OW.pdf]

Supplemental Table S3. Organ weight data. BW = body weight; BRN = brain; OW:BW = (g/g)*100; OW:BRN = g/g.

Organ Weight	Group	n	Male Mean		SD
**Final BW**					
Dosing Day 38	Saline	10	340.42	±	19.138
	Al(OH)3	10	346.67	±	17.604
	RSVpreF	10	334.03	±	23.715
	RSVpreF+Al(OH)3	10	340.75	±	20.741
Recovery Day 28	Saline	5	382.24	±	14.417
	Al(OH)3	5	387.28	±	28.716
	RSVpreF	5	376.54	±	25.218
	RSVpreF+Al(OH)3	5	373.34	±	24.036
**Adrenal**					
Dosing Day 38	Saline	10	0.0649	±	0.0109
	Al(OH)3	10	0.0642	±	0.0057
	RSVpreF	10	0.0645	±	0.0134
	RSVpreF+Al(OH)3	10	0.0617	±	0.0074
Recovery Day 28	Saline	5	0.0663	±	0.0182
	Al(OH)3	5	0.0716	±	0.0101
	RSVpreF	5	0.0578	±	0.0058
	RSVpreF+Al(OH)3	5	0.0691	±	0.0101
**Adrenal:BW**					
Dosing Day 38	Saline	10	0.0191	±	0.0031
	Al(OH)3	10	0.0185	±	0.0014
	RSVpreF	10	0.0194	±	0.0041
	RSVpreF+Al(OH)3	10	0.0181	±	0.0015
Recovery Day 28	Saline	5	0.0173	±	0.0044
	Al(OH)3	5	0.0186	±	0.0029
	RSVpreF	5	0.0154	±	0.0014
	RSVpreF+Al(OH)3	5	0.0186	±	0.0033
**Adrenal:BRN**					
Dosing Day 38	Saline	10	0.0323	±	0.0051
	Al(OH)3	10	0.0327	±	0.0035
	RSVpreF	10	0.0328	±	0.0073
	RSVpreF+Al(OH)3	10	0.0316	±	0.0039
Recovery Day 28	Saline	5	0.0326	±	0.0099
	Al(OH)3	5	0.0359	±	0.0052
	RSVpreF	5	0.0286	±	0.0029
	RSVpreF+Al(OH)3	5	0.0349	±	0.0048
**Brain**					
Dosing Day 38	Saline	10	2.01	±	0.09
	Al(OH)3	10	1.97	±	0.08
	RSVpreF	10	1.97	±	0.07
	RSVpreF+Al(OH)3	10	1.95	±	0.09
Recovery Day 28	Saline	5	2.04	±	0.07
	Al(OH)3	5	2	±	0.07
	RSVpreF	5	2.02	±	0.07
	RSVpreF+Al(OH)3	5	1.98	±	0.13
**Brain:BW**					
Dosing Day 38	Saline	10	0.59	±	0.04
	Al(OH)3	10	0.57	±	0.04
	RSVpreF	10	0.59	±	0.04
	RSVpreF+Al(OH)3	10	0.57	±	0.04
Recovery Day 28	Saline	5	0.53	±	0.03
	Al(OH)3	5	0.52	±	0.05
	RSVpreF	5	0.54	±	0.03
	RSVpreF+Al(OH)3	5	0.53	±	0.03
**Epididymis**					
Dosing Day 38	Saline	10	1.4761	±	0.2184
	Al(OH)3	10	1.459	±	0.1767
	RSVpreF	10	1.4682	±	0.305
	RSVpreF+Al(OH)3	10	1.3319	±	0.1767
Recovery Day 28	Saline	5	1.4635	±	0.2239
	Al(OH)3	5	1.535	±	0.0593
	RSVpreF	5	1.3562	±	0.2055

Organ Weight	Group	n	Female Mean		SD
**Final BW**					
Dosing Day 38	Saline	10	203.15	±	5.731
	Al[OH]3	10	208.01	±	7.135
	RSVpreF	10	204.74	±	7.839
	RSVpreF+Al(OH)3	10	202.53	±	9.105
Recovery Day 27	Saline	5	216.18	±	15.095
	Al[OH]3	5	213.04	±	3.834
	RSVpreF	5	210.9	±	10.354
	RSVpreF+Al(OH)3	5	217.2	±	11.103
**Adrenal**					
Dosing Day 38	Saline	10	0.0785	±	0.011
	Al[OH]3	10	0.0762	±	0.0145
	RSVpreF	10	0.0775	±	0.0162
	RSVpreF+Al(OH)3	10	0.0863	±	0.0089
Recovery Day 27	Saline	5	0.0794	±	0.0152
	Al[OH]3	5	0.0752	±	0.0013
	RSVpreF	5	0.0824	±	0.0046
	RSVpreF+Al(OH)3	5	0.082	±	0.0105
**Adrenal:BW**					
Dosing Day 38	Saline	10	0.0387	±	0.0056
	Al[OH]3	10	0.0368	±	0.0078
	RSVpreF	10	0.0379	±	0.0081
	RSVpreF+Al(OH)3	10	0.0426	±	0.0034
Recovery Day 27	Saline	5	0.037	±	0.0083
	Al[OH]3	5	0.0353	±	0.0007
	RSVpreF	5	0.0391	±	0.0022
	RSVpreF+Al(OH)3	5	0.0379	±	0.0059
**Adrenal:BRN**					
Dosing Day 38	Saline	10	0.0433	±	0.0067
	Al[OH]3	10	0.0421	±	0.0089
	RSVpreF	10	0.0424	±	0.0084
	RSVpreF+Al(OH)3	10	0.0473	±	0.0048
Recovery Day 27	Saline	5	0.0417	±	0.009
	Al[OH]3	5	0.042	±	0.0018
	RSVpreF	5	0.0432	±	0.0029
	RSVpreF+Al(OH)3	5	0.0447	±	0.005
**Brain**					
Dosing Day 38	Saline	10	1.82	±	0.06
	Al[OH]3	10	1.82	±	0.07
	RSVpreF	10	1.83	±	0.06
	RSVpreF+Al(OH)3	10	1.83	±	0.07
Recovery Day 27	Saline	5	1.91	±	0.09
	Al[OH]3	5	1.79	±	0.07
	RSVpreF	5	1.91	±	0.05
	RSVpreF+Al(OH)3	5	1.83	±	0.05
**Brain:BW**					
Dosing Day 38	Saline	10	0.89	±	0.04
	Al[OH]3	10	0.88	±	0.04
	RSVpreF	10	0.89	±	0.04
	RSVpreF+Al(OH)3	10	0.9	±	0.03
Recovery Day 27	Saline	5	0.89	±	0.04
	Al[OH]3	5	0.84	±	0.04
	RSVpreF	5	0.91	±	0.06
	RSVpreF+Al(OH)3	5	0.85	±	0.05
**Heart**					
Dosing Day 38	Saline	10	0.68	±	0.05
	Al[OH]3	10	0.68	±	0.06
	RSVpreF	10	0.72	±	0.04
	RSVpreF+Al(OH)3	10	0.66	±	0.06
Recovery Day 27	Saline	5	0.72	±	0.05
	Al[OH]3	5	0.64	±	0.04
	RSVpreF	5	0.68	±	0.06

	RSVpreF+Al(OH)3	5	1.6496 ± 0.2008
**Epididymis:BW**			
Dosing Day 38	Saline	10	0.4327 ± 0.0502
	Al(OH)3	10	0.4209 ± 0.0478
	RSVpreF	10	0.4388 ± 0.0792
	RSVpreF+Al(OH)3	10	0.3913 ± 0.0496
Recovery Day 28	Saline	5	0.3836 ± 0.0625
	Al(OH)3	5	0.3982 ± 0.0344
	RSVpreF	5	0.3619 ± 0.0645
	RSVpreF+Al(OH)3	5	0.4446 ± 0.0713
**Epididymis:BRN**			
Dosing Day 38	Saline	10	0.7355 ± 0.1156
	Al(OH)3	10	0.7439 ± 0.102
	RSVpreF	10	0.7454 ± 0.161
	RSVpreF+Al(OH)3	10	0.6831 ± 0.0948
Recovery Day 28	Saline	5	0.7199 ± 0.1292
	Al(OH)3	5	0.7689 ± 0.0139
	RSVpreF	5	0.6746 ± 0.1221
	RSVpreF+Al(OH)3	5	0.8359 ± 0.1327
**Heart**			
Dosing Day 38	Saline	10	0.98 ± 0.07
	Al[OH]3	10	1.01 ± 0.1
	RSVpreF	10	0.99 ± 0.09
	RSVpreF+Al(OH)3	10	0.97 ± 0.07
Recovery Day 28	Saline	5	1.1 ± 0.09
	Al[OH]3	5	1.07 ± 0.09
	RSVpreF	5	1.05 ± 0.08
	RSVpreF+Al[OH]3	5	1.12 ± 0.09
**Heart:BW**			
Dosing Day 38	Saline	10	0.29 ± 0.02
	Al[OH]3	10	0.29 ± 0.02
	RSVpreF	10	0.3 ± 0.01
	RSVpreF+Al[OH]3	10	0.28 ± 0.02
Recovery Day 28	Saline	5	0.29 ± 0.01
	Al[OH]3	5	0.28 ± 0.03
	RSVpreF	5	0.28 ± 0.02
	RSVpreF+Al(OH)3	5	0.3 ± 0.02
**Heart:BRN**			
Dosing Day 38	Saline	10	0.49 ± 0.04
	Al[OH]3	10	0.51 ± 0.06
	RSVpreF	10	0.5 ± 0.04
	RSVpreF+Al(OH)3	10	0.5 ± 0.04
Recovery Day 28	Saline	5	0.54 ± 0.05
	Al[OH]3	5	0.54 ± 0.04
	RSVpreF	5	0.52 ± 0.05
	RSVpreF+Al(OH)3	5	0.57 ± 0.03
**Kidney**			
Dosing Day 38	Saline	10	2.3 ± 0.15
	Al[OH]3	10	2.37 ± 0.28
	RSVpreF	10	2.28 ± 0.23
	RSVpreF+Al(OH)3	10	2.27 ± 0.21
	Saline	5	2.55 ± 0.19
Recovery Day 28	Al[OH]3	5	2.57 ± 0.31
	RSVpreF	5	2.51 ± 0.27
	RSVpreF+Al(OH)3	5	2.41 ± 0.11
**Kidney:BW**			
Dosing Day 38	Saline	10	0.68 ± 0.05
	Al[OH]3	10	0.68 ± 0.07
	RSVpreF	10	0.68 ± 0.06
	RSVpreF+Al(OH)3	10	0.67 ± 0.04
Recovery Day 28	Saline	5	0.67 ± 0.06
	Al[OH]3	5	0.66 ± 0.05
	RSVpreF	5	0.67 ± 0.07
	RSVpreF+Al(OH)3	5	0.65 ± 0.05
**Kidney:BRN**			
Dosing Day 38	Saline	10	1.14 ± 0.08

	RSVpreF+Al(OH)3	5	0.92 ± 0.39*
**Heart:BW**			
Dosing Day 38	Saline	10	0.34 ± 0.02
	Al[OH]3	10	0.33 ± 0.03
	RSVpreF	10	0.35 ± 0.02
	RSVpreF+Al(OH)3	10	0.32 ± 0.02
Recovery Day 27	Saline	5	0.33 ± 0.01
	Al[OH]3	5	0.3 ± 0.01
	RSVpreF	5	0.32 ± 0.03
	RSVpreF+Al(OH)3	5	0.43 ± 0.2*
**Heart:BRN**			
Dosing Day 38	Saline	10	0.38 ± 0.03
	Al[OH]3	10	0.37 ± 0.04
	RSVpreF	10	0.39 ± 0.02
	RSVpreF+Al(OH)3	10	0.36 ± 0.03
Recovery Day 27			
	Saline	5	0.38 ± 0.02
	Al[OH]3	5	0.36 ± 0.02
	RSVpreF	5	0.36 ± 0.04
	RSVpreF+Al(OH)3	5	0.51 ± 0.22
**Kidney**			
Dosing Day 38	Saline	10	1.56 ± 0.09
	Al[OH]3	10	1.59 ± 0.13
	RSVpreF	10	1.51 ± 0.11
	RSVpreF+Al(OH)3	10	1.6 ± 0.19
Recovery Day 27	Saline	5	1.57 ± 0.14
	Al[OH]3	5	1.5 ± 0.08
	RSVpreF	5	1.61 ± 0.07
	RSVpreF+Al(OH)3	5	1.49 ± 0.2
**Kidney:BW**			
Dosing Day 38	Saline	10	0.77 ± 0.05
	Al[OH]3	10	0.76 ± 0.05
	RSVpreF	10	0.74 ± 0.07
	RSVpreF+Al(OH)3	10	0.79 ± 0.08
Recovery Day 27	Saline	5	0.73 ± 0.1
	Al[OH]3	5	0.71 ± 0.04
	RSVpreF	5	0.77 ± 0.06
	RSVpreF+Al(OH)3	5	0.68 ± 0.08
**Kidney:BRN**			
Dosing Day 38	Saline	10	0.86 ± 0.06
	Al[OH]3	10	0.88 ± 0.08
	RSVpreF	10	0.83 ± 0.05
	RSVpreF+Al(OH)3	10	0.88 ± 0.09
Recovery Day 27	Saline	5	0.82 ± 0.07
	Al[OH]3	5	0.84 ± 0.04
	RSVpreF	5	0.84 ± 0.03
	RSVpreF+Al(OH)3	5	0.81 ± 0.1
**Liver**			
Dosing Day 38	Saline	10	5.12 ± 0.44
	Al[OH]3	10	5.18 ± 0.32
	RSVpreF	10	5.08 ± 0.3
	RSVpreF+Al(OH)3	10	5.22 ± 0.3
Recovery Day 27	Saline	5	5.27 ± 0.21
	Al[OH]3	5	5.09 ± 0.18
	RSVpreF	5	5.13 ± 0.36
	RSVpreF+Al(OH)3	5	5.32 ± 0.4
**Liver:BW**			
Dosing Day 38	Saline	10	2.52 ± 0.19
	Al[OH]3	10	2.49 ± 0.12
	RSVpreF	10	2.48 ± 0.13
	RSVpreF+Al(OH)3	10	2.58 ± 0.15
Recovery Day 27	Saline	5	2.44 ± 0.15
	Al[OH]3	5	2.39 ± 0.08
	RSVpreF	5	2.43 ± 0.06
	RSVpreF+Al(OH)3	5	2.45 ± 0.09

	Al[OH]3	10	1.21 ± 0.17
	RSVpreF	10	1.16 ± 0.13
	RSVpreF+Al(OH)3	10	1.17 ± 0.14
Recovery Day 28	Saline	5	1.25 ± 0.07
	Al[OH]3	5	1.29 ± 0.17
	RSVpreF	5	1.25 ± 0.16
	RSVpreF+Al(OH)3	5	1.22 ± 0.09
**Liver**			
Dosing Day 38	Saline	10	8.53 ± 0.62
	Al[OH]3	10	8.5 ± 0.74
	RSVpreF	10	8.21 ± 0.83
	RSVpreF+Al(OH)3	10	8.22 ± 1
Recovery Day 28	Saline	5	9.73 ± 0.52
	Al[OH]3	5	9.39 ± 0.91
	RSVpreF	5	9.1 ± 0.56
	RSVpreF+Al(OH)3	5	8.93 ± 0.76
**Liver:BW**			
Dosing Day 38	Saline	10	2.5 ± 0.08
	Al[OH]3	10	2.45 ± 0.14
	RSVpreF	10	2.46 ± 0.14
	RSVpreF+Al(OH)3	10	2.41 ± 0.17
	Saline	5	2.55 ± 0.1
	Al[OH]3	5	2.42 ± 0.11
	RSVpreF	5	2.42 ± 0.11
	RSVpreF+Al(OH)3	5	2.39 ± 0.13
**Liver:BRN**			
Dosing Day 38	Saline	10	4.25 ± 0.41
	Al[OH]3	10	4.34 ± 0.47
	RSVpreF	10	4.17 ± 0.4
	RSVpreF+Al(OH)3	10	4.22 ± 0.55
Recovery Day 28	Saline	5	4.78 ± 0.39
	Al[OH]3	5	4.71 ± 0.49
	RSVpreF	5	4.51 ± 0.26
	RSVpreF+Al(OH)3	5	4.51 ± 0.45
**Prostate**			
Dosing Day 38	Saline	10	1.2219 ± 0.1111
	Al[OH]3	10	1.1919 ± 0.2657
	RSVpreF	10	1.0805 ± 0.2571
	RSVpreF+Al(OH)3	10	1.1541 ± 0.2367
Recovery Day 28	Saline	5	1.1303 ± 0.5163
	Al[OH]3	5	1.1706 ± 0.2524
	RSVpreF	5	1.0692 ± 0.3203
	RSVpreF+Al(OH)3	5	1.2433 ± 0.221
**Prostate:BW**			
Dosing Day 38	Saline	10	0.3599 ± 0.0373
	Al[OH]3	10	0.3431 ± 0.0719
	RSVpreF	10	0.323 ± 0.0754
	RSVpreF+Al(OH)3	10	0.3375 ± 0.0586
Recovery Day 28	Saline	5	0.2944 ± 0.129
	Al[OH]3	5	0.3012 ± 0.0553
	RSVpreF	5	0.2865 ± 0.096
	RSVpreF+Al(OH)3	5	0.3329 ± 0.0546
**Prostate:BRN**			
Dosing Day 38	Saline	10	0.609 ± 0.0632
	Al[OH]3	10	0.6075 ± 0.1376
	RSVpreF	10	0.548 ± 0.1298
Recovery Day 28	RSVpreF+Al(OH)3	10	0.5912 ± 0.1142
	Saline	5	0.559 ± 0.2751
	Al[OH]3	5	0.5881 ± 0.1329
	RSVpreF	5	0.534 ± 0.1767
	RSVpreF+Al(OH)3	5	0.6286 ± 0.1163
**Spleen**			
Dosing Day 38	Saline	10	0.635 ± 0.056
	Al[OH]3	10	0.622 ± 0.071
	RSVpreF	10	0.602 ± 0.092
	RSVpreF+Al(OH)3	10	0.635 ± 0.104

**Liver:BRN**			
Dosing Day 38	Saline	10	2.82 ± 0.25
	Al[OH]3	10	2.85 ± 0.17
	RSVpreF	10	2.78 ± 0.19
	RSVpreF+Al(OH)3	10	2.86 ± 0.14
Recovery Day 27	Saline	5	2.76 ± 0.2
	Al[OH]3	5	2.84 ± 0.11
	RSVpreF	5	2.69 ± 0.22
	RSVpreF+Al(OH)3	5	2.9 ± 0.23
**Ovary**			
Dosing Day 38	Saline	10	0.1274 ± 0.0245
	Al[OH]3	10	0.1151 ± 0.0204
	RSVpreF	10	0.1113 ± 0.0203
	RSVpreF+Al(OH)3	10	0.1138 ± 0.0243
Recovery Day 27	Saline	5	0.1098 ± 0.0338
	Al[OH]3	5	0.0938 ± 0.0142
	RSVpreF	5	0.1242 ± 0.0245
	RSVpreF+Al(OH)3	5	0.0958 ± 0.0273
**Ovary:BW**			
Dosing Day 38	Saline	10	0.0627 ± 0.0116
	Al[OH]3	10	0.0553 ± 0.0094
	RSVpreF	10	0.0545 ± 0.0102
	RSVpreF+Al(OH)3	10	0.056 ± 0.0109
Recovery Day 27	Saline	5	0.0511 ± 0.0161
	Al[OH]3	5	0.044 ± 0.0067
	RSVpreF	5	0.0588 ± 0.0111
	RSVpreF+Al(OH)3	5	0.0439 ± 0.0114
**Ovary:BRN**			
Dosing Day 38	Saline	10	0.0702 ± 0.0137
	Al[OH]3	10	0.0632 ± 0.0106
	RSVpreF	10	0.061 ± 0.0111
	RSVpreF+Al(OH)3	10	0.0623 ± 0.0129
Recovery Day 27	Saline	5	0.0576 ± 0.0185
	Al[OH]3	5	0.0525 ± 0.0088
	RSVpreF	5	0.0653 ± 0.0142
	RSVpreF+Al(OH)3	5	0.0522 ± 0.0145
**Spleen**	Dosing Day 38		
	Saline	10	0.428 ± 0.084
	Al[OH]3	10	0.497 ± 0.06
	RSVpreF	10	0.427 ± 0.042
	RSVpreF+Al(OH)3	10	0.492 ± 0.073
Recovery Day 27	Saline	5	0.437 ± 0.061
	Al[OH]3	5	0.389 ± 0.038
	RSVpreF	5	0.437 ± 0.112
	RSVpreF+Al(OH)3	5	0.475 ± 0.058
**Spleen:BW**	Dosing Day 38		
	Saline	10	0.21 ± 0.039
	Al[OH]3	10	0.239 ± 0.027
	RSVpreF	10	0.208 ± 0.017
	RSVpreF+Al(OH)3	10	0.243 ± 0.036
Recovery Day 27	Saline	5	0.203 ± 0.028
	Al[OH]3	5	0.183 ± 0.017
	RSVpreF	5	0.208 ± 0.055
	RSVpreF+Al(OH)3	5	0.218 ± 0.018
**Spleen:BRN**	Dosing Day 38		
	Saline	10	0.236 ± 0.044
	Al[OH]3	10	0.273 ± 0.03
	RSVpreF	10	0.234 ± 0.023
	RSVpreF+Al(OH)3	10	0.269 ± 0.036
Recovery Day 27	Saline	5	0.229 ± 0.037
	Al[OH]3	5	0.217 ± 0.018
	RSVpreF	5	0.229 ± 0.058
	RSVpreF+Al(OH)3	5	0.259 ± 0.033
**Thymus**			
Dosing Day 38	Saline	10	0.2807 ± 0.0479
	Al[OH]3	10	0.2872 ± 0.0519

Recovery Day 28	Saline	5	0.694 ± 0.107
	Al[OH]3	5	0.641 ± 0.08
	RSVpreF	5	0.612 ± 0.043
	RSVpreF+Al(OH)3	5	0.649 ± 0.14
**Spleen:BW**			
Dosing Day 38	Saline	10	0.187 ± 0.018
	Al[OH]3	10	0.18 ± 0.022
	RSVpreF	10	0.18 ± 0.025
	RSVpreF+Al(OH)3	10	0.186 ± 0.023
Recovery Day 28	Saline	5	0.182 ± 0.027
	Al[OH]3	5	0.166 ± 0.022
	RSVpreF	5	0.163 ± 0.015
	RSVpreF+Al(OH)3	5	0.174 ± 0.036
**Spleen:BRN**			
Dosing Day 38	Saline	10	0.316 ± 0.032
	Al[OH]3	10	0.317 ± 0.037
	RSVpreF	10	0.305 ± 0.047
	RSVpreF+Al(OH)3	10	0.326 ± 0.054
Recovery Day 28	Saline	5	0.34 ± 0.044
	Al[OH]3	5	0.32 ± 0.031
	RSVpreF	5	0.303 ± 0.018
	RSVpreF+Al(OH)3	5	0.326 ± 0.062
**Testis**			
Dosing Day 38	Saline	10	3.638 ± 0.24
	Al[OH]3	10	3.865 ± 0.28
	RSVpreF	10	3.622 ± 0.365
	RSVpreF+Al(OH)3	10	3.654 ± 0.293
Recovery Day 28	Saline	5	3.825 ± 0.211
	Al[OH]3	5	3.81 ± 0.091
	RSVpreF	5	3.536 ± 0.297
	RSVpreF+Al(OH)3	5	3.682 ± 0.266
**Testis:BW**			
Dosing Day 38	Saline	10	1.07 ± 0.061
	Al[OH]3	10	1.117 ± 0.088
	RSVpreF	10	1.085 ± 0.093
	RSVpreF+Al(OH)3	10	1.073 ± 0.061
Recovery Day 28	Saline	5	1.001 ± 0.044
	Al[OH]3	5	0.988 ± 0.082
	RSVpreF	5	0.939 ± 0.048
	RSVpreF+Al(OH)3	5	0.992 ± 0.124
**Testis:BRN**			
Dosing Day 38	Saline	10	1.81 ± 0.097
	Al[OH]3	10	1.969 ± 0.171
	RSVpreF	10	1.836 ± 0.172
	RSVpreF+Al(OH)3	10	1.877 ± 0.206
Recovery Day 28	Saline	5	1.879 ± 0.168
	Al[OH]3	5	1.91 ± 0.085
	RSVpreF	5	1.75 ± 0.125
	RSVpreF+Al(OH)3	5	1.863 ± 0.195
**Thymus**			
Dosing Day 38	Saline	10	0.4272 ± 0.0556
	Al[OH]3	10	0.4382 ± 0.0741
	RSVpreF	10	0.3789 ± 0.0626
	RSVpreF+Al(OH)3	10	0.4198 ± 0.0574
Recovery Day 28	Saline	5	0.3656 ± 0.0364
	Al[OH]3	5	0.4805 ± 0.0677
	RSVpreF	5	0.3646 ± 0.0836
	RSVpreF+Al(OH)3	5	0.3666 ± 0.0476
**Thymus:BW**			
Dosing Day 38	Saline	10	0.1258 ± 0.0175
	Al[OH]3	10	0.1267 ± 0.0233
	RSVpreF	10	0.1142 ± 0.021
	RSVpreF+Al(OH)3	10	0.1234 ± 0.0163
Recovery Day 28	Saline	5	0.0958 ± 0.0106
	Al[OH]3	5	0.1243 ± 0.0173
	RSVpreF	5	0.0974 ± 0.0254
	RSVpreF	10	0.278 ± 0.0551
	RSVpreF+Al(OH)3	10	0.2836 ± 0.0607
Recovery Day 27	Saline	5	0.232 ± 0.0554
	Al[OH]3	5	0.2558 ± 0.0259
	RSVpreF	5	0.2628 ± 0.0453
	RSVpreF+Al(OH)3	5	0.2683 ± 0.092
**Thymus:BW**			
Dosing Day 38	Saline	10	0.1379 ± 0.0218
	Al[OH]3	10	0.138 ± 0.024
	RSVpreF	10	0.1358 ± 0.0264
	RSVpreF+Al(OH)3	10	0.1399 ± 0.0279
Recovery Day 27	Saline	5	0.1073 ± 0.0253
	Al[OH]3	5	0.1202 ± 0.0137
	RSVpreF	5	0.1243 ± 0.0181
	RSVpreF+Al(OH)3	5	0.1234 ± 0.0403
**Thymus:BRN**			
Dosing Day 38	Saline	10	0.1543 ± 0.0237
	Al[OH]3	10	0.158 ± 0.0287
	RSVpreF	10	0.1527 ± 0.0327
	RSVpreF+Al(OH)3	10	0.155 ± 0.03
Recovery Day 27	Saline	5	0.1206 ± 0.0253
	Al[OH]3	5	0.1428 ± 0.0139
	RSVpreF	5	0.1378 ± 0.0249
	RSVpreF+Al(OH)3	5	0.1457 ± 0.048

	RSVpreF+Al(OH)3	5	0.0982 ± 0.0103
**Thymus:BRN**			
Dosing Day 38	Saline	10	0.213 ± 0.0302
	Al[OH]3	10	0.2235 ± 0.0399
	RSVpreF	10	0.1925 ± 0.0331
	RSVpreF+Al(OH)3	10	0.2159 ± 0.037
Recovery Day 28	Saline	5	0.1793 ± 0.0182
	Al[OH]3	5	0.2407 ± 0.0345
	RSVpreF	5	0.1818 ± 0.0479
	RSVpreF+Al(OH)3	5	0.1847 ± 0.0193
