# Supplementary material for: Favorable Nonclinical Safety Profile of RSVpreF Bivalent Vaccine in Rats and Rabbits
Source: Vaccines (Basel). 2024 Dec 31;13(1):26. doi: 10.3390/vaccines13010026 (PMC11769190; doi:10.3390/vaccines13010026)
Supplement: Supplementary file 1 [file vaccines-13-00026-s001.zip › Supplemental Tables S4_S6.pdf]

Supplemental Table S4. Summary of rabbit maternal body weight and food consumption data during the pre-mating, gestation, and lactation periods, respectively. PF-07203723 = RSVpreF bivalent vaccine.

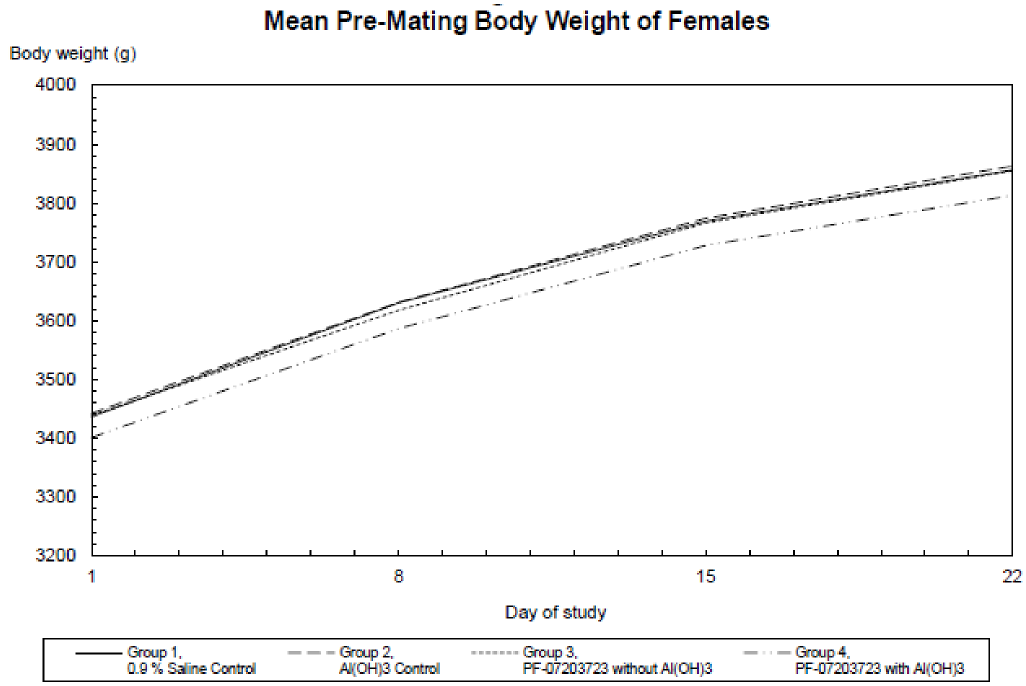

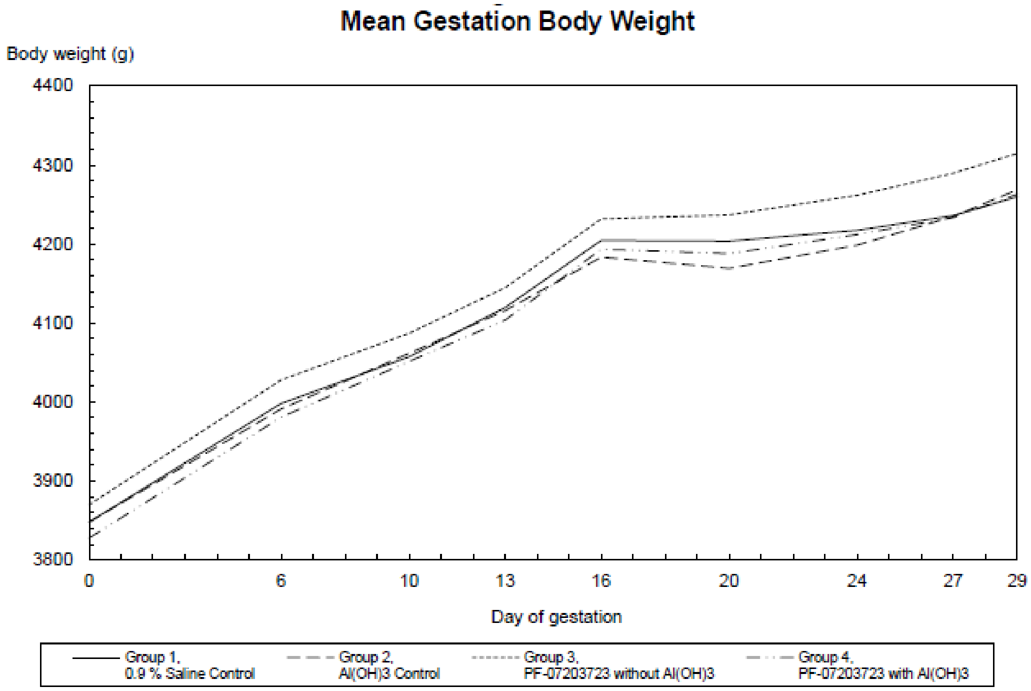

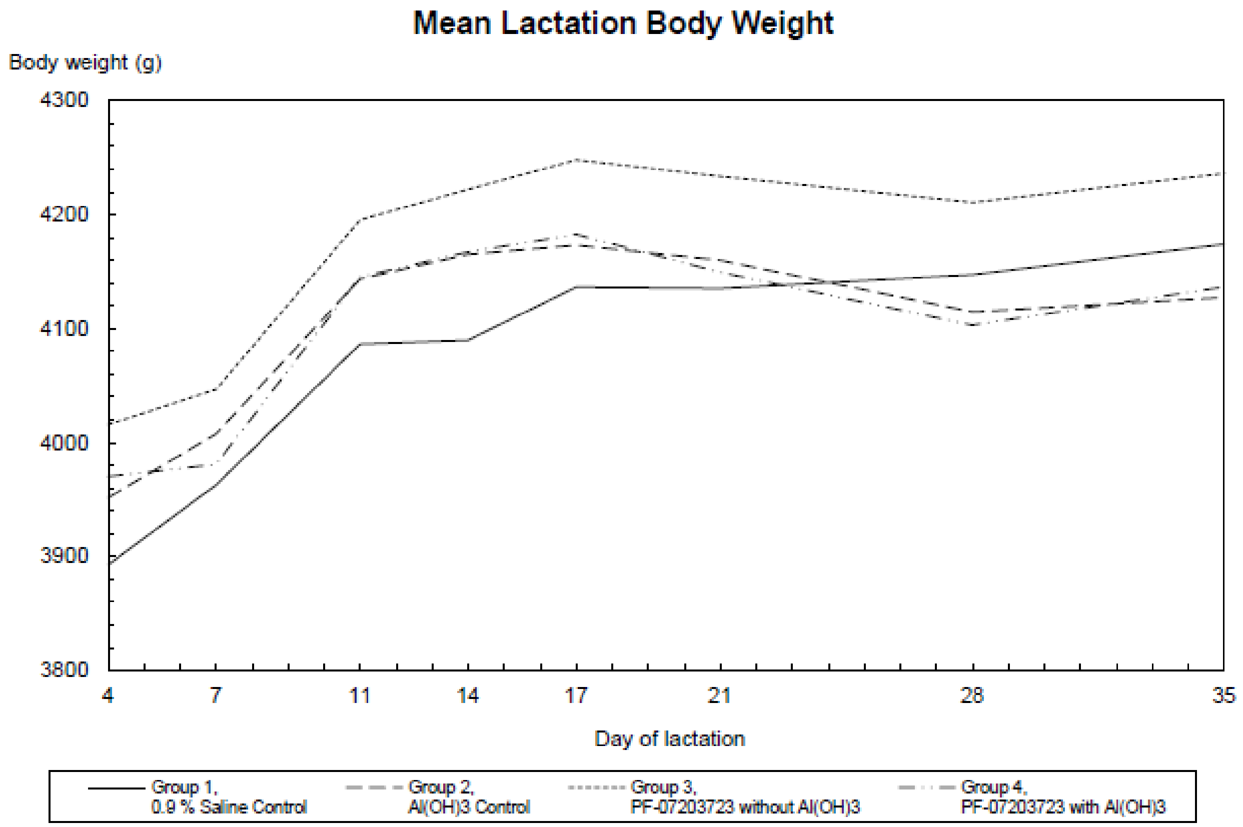
Mean Lactation Body Weight
Body weight (g)
4300
4200
4100
4000
3900
3800
4
7
11
14
17
21
28
35
Day of lactation
Group 1,
0.9 % Saline Control
Group 2,
Al(OH)3 Control
Group 3,
PF-07203723 without Al(OH)3
Group 4,
PF-07203723 with Al(OH)3

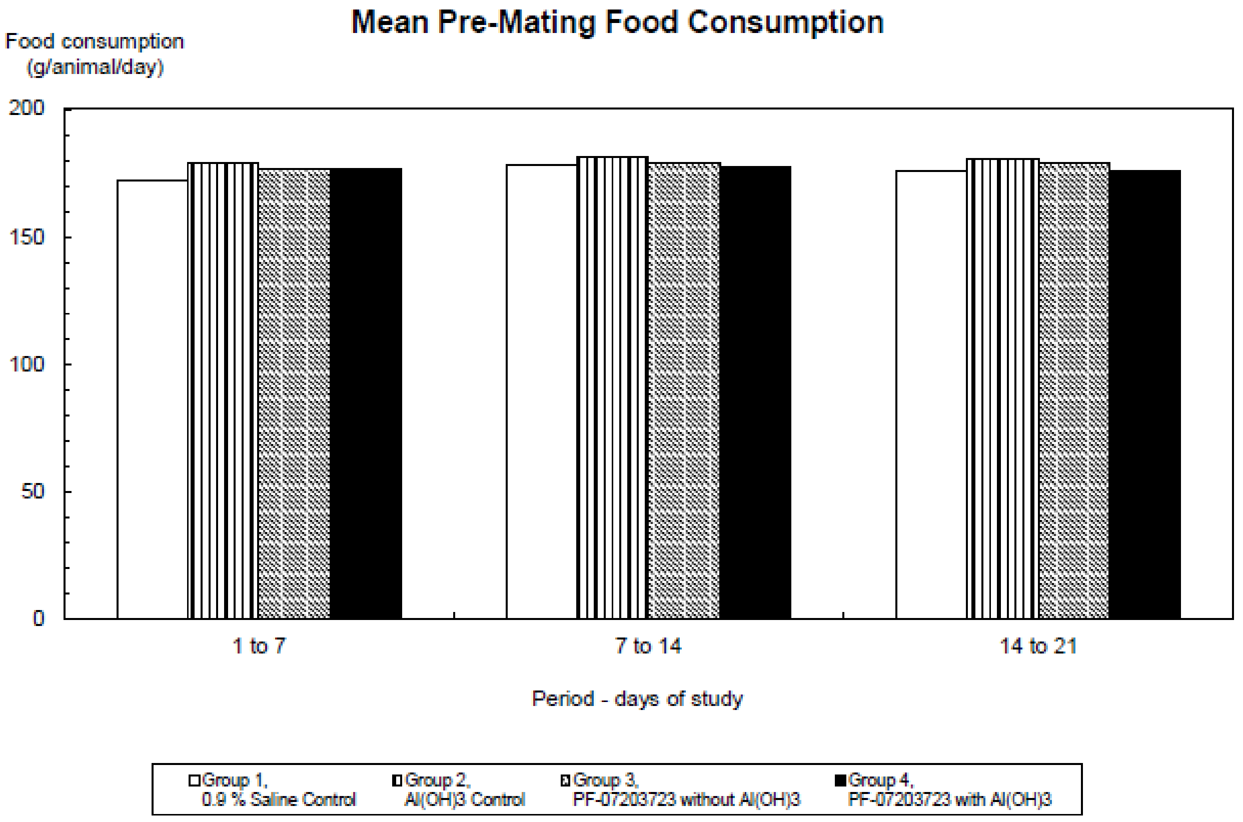
Mean Pre-Mating Food Consumption
Food consumption
(g/animal/day)
200
150
100
50
0
1 to 7
7 to 14
14 to 21
Period - days of study
Group 1,
0.9 % Saline Control
Group 2,
Al(OH)3 Control
Group 3,
PF-07203723 without Al(OH)3
Group 4,
PF-07203723 with Al(OH)3

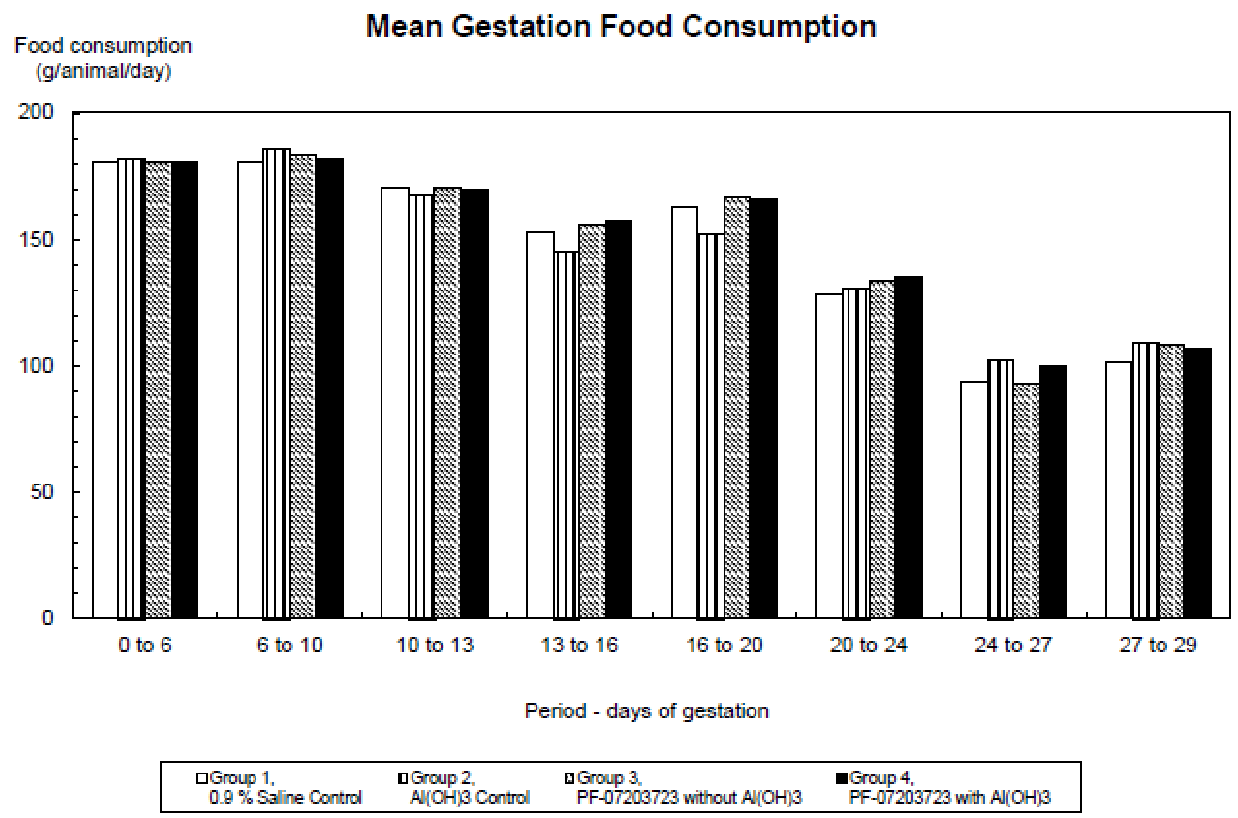
Mean Gestation Food Consumption
Food consumption (g/animal/day)
200
150
100
50
0
0 to 6
6 to 10
10 to 13
13 to 16
16 to 20
20 to 24
24 to 27
27 to 29
Period - days of gestation
Group 1, 0.9 % Saline Control
Group 2, Al(OH)3 Control
Group 3, PF-07203723 without Al(OH)3
Group 4, PF-07203723 with Al(OH)3

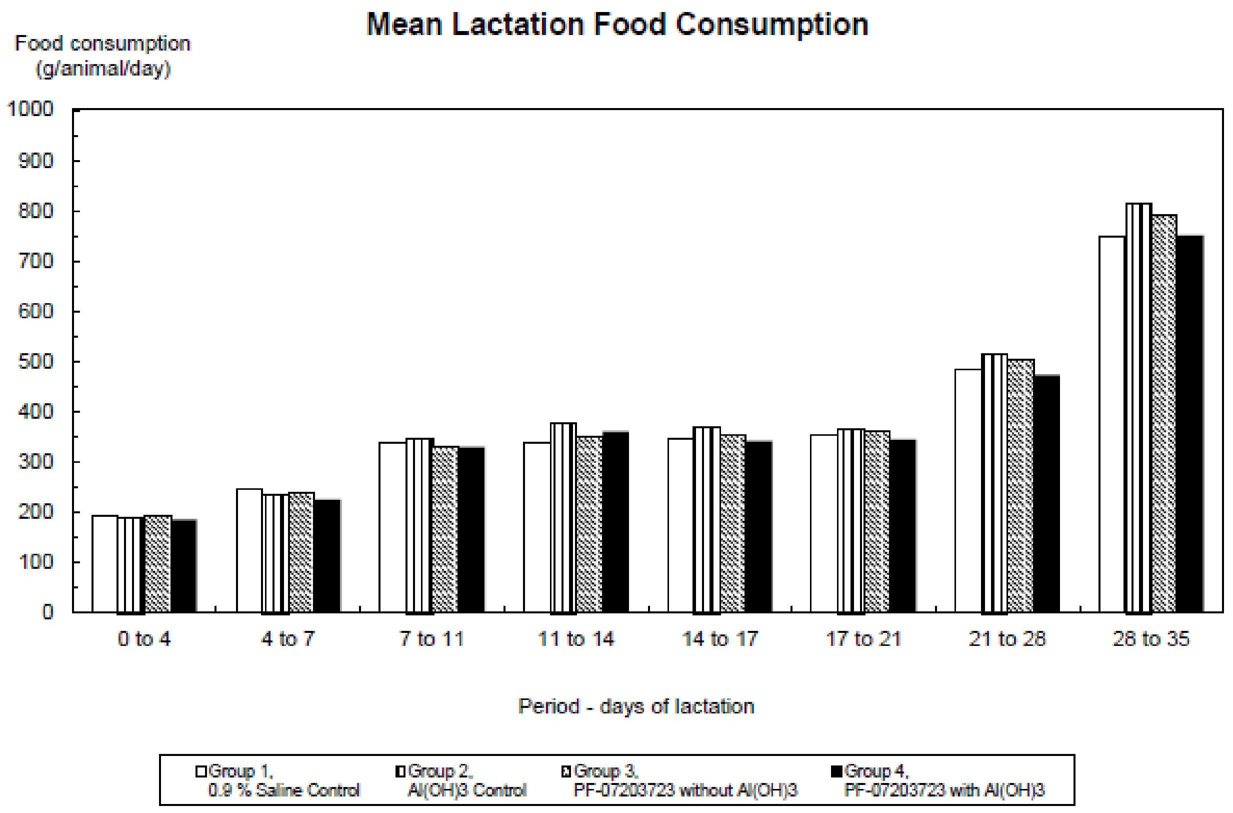
Mean Lactation Food Consumption
Food consumption (g/animal/day)
1000
900
800
700
600
500
400
300
200
100
0
0 to 4
4 to 7
7 to 11
11 to 14
14 to 17
17 to 21
21 to 28
28 to 35
Period - days of lactation
Group 1, 0.9 % Saline Control
Group 2, Al(OH)3 Control
Group 3, PF-07203723 without Al(OH)3
Group 4, PF-07203723 with Al(OH)3

Supplemental Table S5. Summary of rabbit fetal examination data from the embryo fetal development study with RSVpreF and RSVpreF + $Al(OH)_3$ (n = 18-20 rabbits per group). Incidence data are presented as litter/fetus and numbers in parentheses following litter and fetal incidence indicate the percent of fetuses affected. M = malformation; V = variation; A = anomaly.

	Saline	Vehicle $Al(OH)_3$	RSVpreF	RSVpreF + $Al(OH)_3$
**External**				
Number Examined Litter/Fetus	*20/183*	*18/166*	*20/175*	*19/152*
Paw, Hyperflexion - [M]	0/0 (0)	0/0 (0)	0/0 (0)	1/1 (0.7)
Tail, Acaudia - [M]	1/1 (0.5)	0/0 (0)	0/0 (0)	0/0 (0)
**Visceral**				
Number Examined Litter/Fetus - Head	*20/88*	*18/78*	*20/82*	*19/72*
Eye, Retina, Fold – [M]	0/0 (0)	1/1 (1.3)	0/0 (0)	0/0 (0)
Buccal cavity, Cleft palate – [M]	0/0 (0)	0/0 (0)	0/0 (0)	1/1 (1.4)
Number Examined Litter/Fetus - Body	*20/183*	*18/166*	*20/175*	*19/152*
Abdomen, Fluid-filled - [A]	0/0 (0)	0/0 (0)	0/0 (0)	1/1 (0.7)
Gallbladder, Absent - [M]	1/1 (0.5)	0/0 (0)	0/0 (0)	1/1 (0.7)
Gallbladder, Small - [A]	0/0 (0)	0/0 (0)	2/2 (1.1)	1/1 (0.7)
Gallbladder, Supernumerary - [A]	1/1 (0.5)	0/0 (0)	0/0 (0)	0/0 (0)
Heart, Aortic valve, Absent - [M]	0/0 (0)	0/0 (0)	1/1 (0.6)	0/0 (0)
Heart, Ventricular septum defect - [M]	2/2 (1.1)	2/2 (1.2)	1/1 (0.6)	0/0 (0)
Heart, Pulmonary valve, Absent - [M]	2/2 (1.1)	1/1 (0.6)	0/0 (0)	0/0 (0)
Heart, Ventricle, Small - [M]	0/0 (0)	0/0 (0)	1/1 (0.6)	0/0 (0)
Kidney, Dilated renal pelvis - [A]	0/0 (0)	1/1 (0.6)	1/1 (0.6)	0/0 (0)
Kidney, Malpositioned – [A]	1/1 (0.5)	0/0 (0)	0/0 (0)	2/2 (1.3)
Kidney, Misshapen – [A]	0/0 (0)	0/0 (0)	0/0 (0)	2/2 (1.3)
Liver, Lobe, Cyst – [A]	0/0 (0)	0/0 (0)	0/0 (0)	1/1 (0.7)
Lung, Lobe, Absent – [A]	7/11 (6.0)	4/5 (3.0)	2/8 (4.6)	1/1 (0.7)
Lung, Lobe, Cyst – [A]	0/0 (0)	0/0 (0)	1/1 (0.6)	1/1 (0.7)
Lung, Abnormal lobation - [M]	0/0 (0)	0/0 (0)	1/1 (0.6)	0/0 (0)
Major blood vessel, Aortic arch, Dilated – [M]	2/2 (1.1)	2/2 (1.2)	1/1 (0.6)	0/0 (0)
Major blood vessel, Aortic arch, Interrupted – [M]	0/0 (0)	0/0 (0)	1/1 (0.6)	0/0 (0)
Major blood vessel, Common carotid trunk, Absent – [V]	14/45 (24.6)	13/50 (30.1)	17/45 (25.7)	12/39 (25.7)
Major blood vessel, Innominate artery, Absent – [V]	0/0 (0)	0/0 (0)	2/2 (1.1)	0/0 (0)
Major blood vessel, Pulmonary trunk, Atresia – [M]	2/2 (1.1)	1/1 (0.6)	0/0 (0)	0/0 (0)
Major blood vessel, Subclavian artery, Malpositioned – [A]	0/0 (0)	1/1 (0.6)	1/1 (0.6)	0/0 (0)
Major blood vessel, Subclavian artery, Retroesophageal – [A]	0/0 (0)	0/0 (0)	1/1 (0.6)	1/1 (0.7)
Ovary, Cyst - [A]	0/0 (0)	2/2 (2.4)	0/0 (0)	1/1 (1.1)
Ovary, Malpositioned – [A]	0/0 (0)	0/0 (0)	0/0 (0)	1/1 (1.1)
Testis, Absent - [M]	1/1 (1.0)	0/0 (0)	0/0 (0)	0/0 (0)

Testis, Malpositioned – [M]	0/0 (0)	0/0 (0)	0/0 (0)	1/1 (1.6)
Ureter, Convoluted - [A]	1/1 (0.5)	0/0 (0)	0/0 (0)	0/0 (0)
Ureter, Retrocaval - [A]	8/9 (4.9)	6/8 (4.8)	4/5 (2.9)	5/6 (3.9)
Ureter, Short - [A]	0/0 (0)	0/0 (0)	0/0 (0)	1/1 (0.7)
Vein, Azygos vein, Transposed – [A]	1/2 (1.1)	0/0 (0)	0/0 (0)	0/0 (0)
**Skeletal**				
Number Examined Litter/Fetus - Head	*20/95*	*18/88*	*20/93*	*19/80*
Skull, Cranium, Sutural bone [A]	0/0 (0)	2/2 (2.3)	1/1 (1.1)	1/1 (1.3)
Skull, Fontanelle, Large – [A]	1/1 (1.1)	0/0 (0)	0/0 (0)	0/0 (0)
Skull, Fontanelle, Small - [A]	0/0 (0)	2/5 (5.7)	1/1 (1.1)	0/0 (0)
Skull, Hyoid, Misshapen - [A]	0/0 (0)	1/1 (1.1)	0/0 (0)	1/1 (1.3)
Skull, Nasal, Unossified line – [A]	0/0 (0)	1/1 (1.1)	0/0 (0)	0/0 (0)
Skull, Parietal, Unossified line – [A]	0/0 (0)	0/0 (0)	0/0 (0)	1/1 (1.3)
Skull, Presphenoid, incomplete ossification - [A]	1/1 (1.1)	0/0 (0)	0/0 (0)	0/0 (0)
Number Examined Litter/Fetus - Body	*20/183*	*18/166*	*20/175*	*19/152*
Forepaw, Metacarpal, Incomplete ossification, 2nd to 5th digit – [A]	1/1 (0.5)	0/0 (0)	0/0 (0)	0/0 (0)
Forepaw, Metacarpal, Unossified, 1st digit – [V]	1/1 (0.5)	0/0 (0)	1/1 (0.6)	2/2 (1.3)
Forepaw, Phalanx, Incomplete ossification, proximal – [A]	1/1 (0.5)	0/0 (0)	0/0 (0)	1/2 (1.3)
Forepaw, Phalanx, Unossified, middle – [V]	0/0 (0)	0/0 (0)	0/0 (0)	1/2 (1.3)
General, Vertebrae, Multiple abnormalities – [M]	1/1 (0.5)	0/0 (0)	0/0 (0)	0/0 (0)
Hindpaw, Tarsal bone, Unossified – [A]	0/0 (0)	0/0 (0)	0/0 (0)	1/1 (0.7)
Pectoral girdle, Scapula, Misshapen – [A]	0/0 (0)	0/0 (0)	0/0 (0)	1/1 (0.7)
Pelvic girdle, Malpositioned – [A]	6/8 (4.4)	8/12 (7.2)	6/7 (4.0)	4/5 (3.3)
Pubis, Incomplete ossification - [A]	2/3 (1.6)	1/1 (0.6)	0/0 (0)	2/2 (1.3)
Ribs, Detached – [A]	6/7 (3.8)	4/6 (3.6)	5/6 (3.4)	1/1 (0.7
Ribs, Interrupted – [A]	1/1 (0.5)	0/0 (0)	0/0 (0)	0/0 (0)
Ribs, Misshapen – [A]	0/0 (0)	0/0 (0)	1/1 (0.6)	0/0 (0)
Ribs, Nodulated – [A]	1/1 (0.5)	0/0 (0)	0/0 (0)	0/0 (0)
Ribs, Number of full ribs = 12/12 – [V]	20/111 (60.7)	17/96 (57.8)	19/103 (58.9)	18/92 (60.5)
Ribs, Number of full ribs = 12/13 – [V]	13/24 (13.1)	14/26 (15.7)	14/23 (13.1)	10/21 (13.8)
Ribs, Short –[A]	9/15 (8.2)	13/15 (9.0)	11/17 (9.7)	9/15 (9.9)
Ribs, Supernumerary cervical – [A]	0/0 (0)	3/4 (2.4)	3/7 (4.0)	2/3 (2.0)
Ribs, Supernumerary lumbar – [A]	15/28 (15.3)	11/24 (14.5)	17/33 (18.9)	12/23 (15.1)
Sternebra, Asymmetic – [A]	2/3 (1.6)	1/1 (0.6)	0/0 (0)	0/0 (0)
Sternebra, Extra ossification site – [A]	1/1 (0.5)	0/0 (0)	1/1 (0.6)	2/2 (1.3)
Sternebra, Fused – [M]	2/2 (1.1)	1/2 (1.2)	0/0 (0)	1/1 (0.7)
Sternebra, Incomplete ossification, 1st/3rd – [A]	1/1 (0.5)	0/0 (0)	0/0 (0)	0/0 (0)
Sternebra, Minor fusion – [A]	2/2 (1.1)	2/5 (3.0)	3/3 (1.7)	0/0 (0)
Sternebra, Misshapen – [A]	0/0 (0)	0/0 (0)	0/0 (0)	1/1 (0.7)
Sternebra, Unossified, 5th – [V]	11/23 (12.6)	10/27 (16.3)	7/17 (9.7)	7/18 (11.8)

Sternebra, Unossified, 6th [V]	4/7 (3.8)	2/2 (1.2)	0/0 (0)	2/3 (2.0)
Sternebra, Incomplete ossification, 2nd/4th – [V]	3/3 (1.6)	0/0 (0)	1/1 (0.6)	1/1 (0.7)
Sternebra, Incomplete ossification, 6th – [V]	7/9 (4.9)	4/4 (2.4)	5/13 (7.4)	6/11 (7.2)
Vertebra, Caudal, Malpositioned – [A]	1/1 (0.5)	1/1 (0.6)	0/0 (0)	2/2 (1.3)
Vertebra, Caudal, Misshapen – [A]	0/0 (0)	0/0 (0)	1/1 (0.6)	1/1 (0.7)
Vertebra, Caudal, Number <14 – [A]	0/0 (0)	0/0 (0)	1/1 (0.6)	1/1 (0.7)
Vertebra, Cervical, Fused centrum - [A]	0/0 (0)	0/0 (0)	1/1 (0.6)	0/0 (0)
Vertebra, Cervical, Small centrum – [A]	0/0 (0)	0/0 (0)	1/1 (0.6)	0/0 (0)
Vertebra, Lumbar, Number = 6 – [V]	14/48 (26.2)	14/32 (19.3)	15/31 (17.7)	12/35 (23.0)
Vertebra, Lumbar, Number = 8 – [V]	3/3 (1.6)	4/7 (4.2)	3/4 (2.3)	2/4 (2.6)
Vertebra, Thoracic, Incomplete ossification of centrum, 1st to 9th [A]	0/0 (0)	1/1/ (0.6)	0/0 (0)	1/1 (0.7)
Vertebra, Thoracic, Multiple abnormalities – [M]	0/0 (0)	0/0 (0)	2/2 (1.1)	0/0 (0)
Vertebra, Thoracic, Number = 12 – [V]	20/111 (60.7)	17/96 (57.8)	19/103 (58.9)	18/92 (60.5)

Supplemental Table S6. Summary of rabbit pre-weaning functional reflexes at end of pre-weaning period (postnatal day 35).

	**Saline**	**Vehicle $Al(OH)_3$**	**RSVpreF**	**RSVpreF + $Al(OH)_3$**
Pupillary Reflex (% animals positive)	100	100	100	100
Auditory Reflex (% animals positive)	100	100	100	100
